# A Novel Rat Model to Test Intra-Abdominal Anti-adhesive Therapy

**DOI:** 10.3389/fsurg.2020.00012

**Published:** 2020-04-08

**Authors:** Rajan Sundaresan Vediappan, Catherine Bennett, Ahmed Bassiouni, Matthew Smith, John Finnie, Markus Trochsler, Alkis J. Psaltis, Sarah Vreugde, Peter J. Wormald

**Affiliations:** ^1^Department of Surgery - Otolaryngology Head and Neck Surgery, The University of Adelaide, Adelaide, SA, Australia; ^2^The Queen Elizabeth Hospital, Animal Experiment Suit, Adelaide, SA, Australia; ^3^SA Pathology and Adelaide Medical School, The University of Adelaide, Adelaide, SA, Australia; ^4^Department of Surgery, The University of Adelaide, Adelaide, SA, Australia

**Keywords:** Kaolin, abdominal adhesion, animal model, fibrosis, anti-adhesive agent

## Abstract

**Background:** Adhesion formation after abdominal surgery is considered almost inevitable and a major cause of morbidity. Novel treatments have been proposed, however there is a lack of suitable small animal models for pre-clinical evaluation, mainly due to inconsistency in adhesion formation in positive control animals. Here, we propose a new rat model of abdominal adhesions using Kaolin as the adhesion-inducing agent at an optimized dosage for testing newer agents in respect to their anti-adhesive property.

**Materials and Methods:** Twenty-five adult (8–10 week old) male Wistar albino rats underwent midline laparotomy and caecal abrasion and were randomized to receive topical applications of normal saline or different concentrations and volumes of a Kaolin-based formulation. At day 14 rats were humanely killed, and adhesions graded macroscopically by an investigator blinded to the treatment groups, using pre-determined adhesion scores and microscopically using histopathology.

**Results:** Kaolin at 0.005 g/mL caused consistent adhesions without compromising rat viability. At higher doses significant morbidity and mortality was observed in the animals treated.

**Conclusions:** Kaolin induced adhesion in a rat abdominal surgery model is reliable and can be safely used to test the efficacy of novel anti-adhesive formulations to prevent intra-abdominal adhesions.

## Introduction

Scarring or fibrosis is an inevitable manifestation of the wound healing process in the human body after surgery. This often results in undesirable outcomes. Scarring after abdominal surgery often results in the formation of adhesions where scar tissue connects organs with each other, often resulting in post-surgical morbidity. Around 7 million open abdominal surgeries occur each year in the US and Europe with adhesions estimated in up to 90% of cases, costing the USA health care system $USD 2.3 billion annually (1). Postsurgical adhesions are the largest single cause of intestinal obstruction with a mortality rate of 10% and can also contribute to female infertility (2–5). Numerous strategies have been recommended to prevent peritoneal adhesions; however, none are widely adopted due to poor efficacy or risk of adverse events (6). It is essential to have an animal model that can be used to test novel anti-adhesive strategies in abdominal surgery and to test substances and strategies to prevent adhesions. To date although several pro-adhesion models exists, they lack consistency of adhesion formation and therefore a better animal model is required.

Kaolin is known to induce inflammation and foreign body reactions and has been used to induce adhesions in animal models, especially in pulmonary fibrosis (7), hepatic fibrosis (8), and subarachnoid dural adhesion clinical models (9). This study tested the dose-dependent effects of kaolin to induce adhesions in a rat colon abrasion model.

## Materials and Methods

The University of Adelaide and Central Adelaide Local Health Network/SA Pathology Animal Ethics Committees (AEC) approved the study to be conducted at The Queen Elizabeth Hospital Experimental Surgical Suite (The University of Adelaide AEC M-2017-061 and CALHN/SA Pathology AEC 25-17).

### Animals and Materials

Male Wistar albino rats were purchased from Laboratory Animal Services Medical School (The University of Adelaide, SA, Australia), 8 to 10 weeks old, with an average weight between 350 and 500 grams. Rats were housed 1 week prior to surgery under standard laboratory conditions (temperature 21°C ± 2°C, humidity 55% ± 10%, 12:12 h light-dark-cycle). Rats were housed in groups of 3 per cage and food and water were provided in a standard manner. Kaolin [Aluminum silicate Hydroxide, Al_2_Si_2_O_5_(OH)_4_] was purchased from Sigma-Aldrich, St. Louis, Missouri, United States.

### Surgical Procedure

Surgical procedures were performed by the same surgeons (RSV, CB) after a period of training and a maximum group size of five animals per day was used to ensure close monitoring during the immediate postoperative period. Anesthesia was achieved using a sealed chamber to deliver 2–3% Isoflurane after which the animal was positioned for surgery in supine position and anesthesia maintained with isoflurane over an open mask. Analgesia was provided pre-operatively by subcutaneous injection of Buprenorphine (0.05 mg/kg) and post-operative 8 hourly for 48 h. The surgery was conducted in aseptic manner and a prophylactic dosage of broad-spectrum antibiotic in the form of Amoxicillin Clavulanic acid 5 mg/kg (Clavulox^*^ Zoetis Australia, Rhodes, NSW, Australia) was also administered via subcutaneous injection.

**Figure 1:**
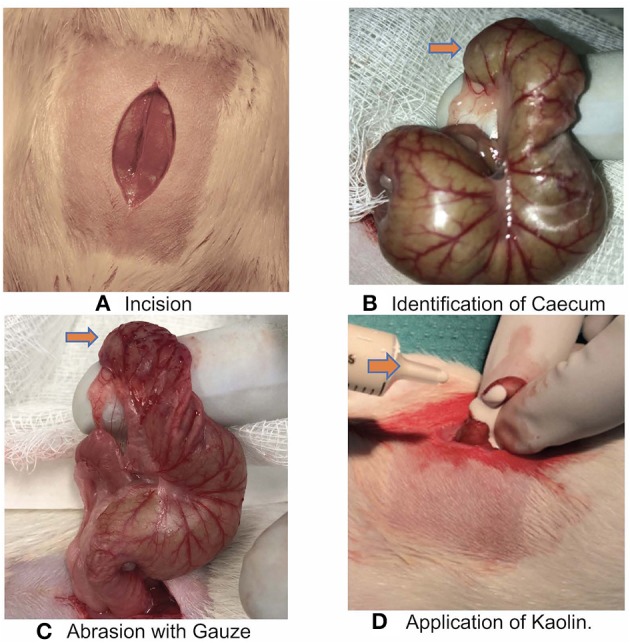
**(A)** Incision over the Rat abdominal wall after preparation. **(B)** Identification of Caecum (orange arrow). **(C)** Abrasion over the caecum with gauze till bleeding spots appear (orange arrow). **(D)** Application of Kaolin over the abrasion/enterotomy (orange arrow).

Rats then underwent a laparotomy and a colon abrasion (9) or a colon abrasion with enterotomy. Briefly, the abdomen was shaved and prepared with alcohol and after drying, a 3 cm laparotomy was performed to gain access to the abdominal cavity (Figure 1A). In the caecal abrasion group, the caecum was delivered (Figure 1B) and kept moist with saline-soaked gauze whilst a dry gauze was used to rub the caecum repeatedly until sub serosal bleeding occurred over an area of 1 cm^2^ (Figure 1C). The caecum was then returned to the abdomen and the abdominal wall closed in layers with a 3-0 Polyglactin suture. Prior to the placement of the final abdominal closure suture, rats were randomized to receive the following treatments:

1. 4 mL normal saline, *n* = 5
2. 4 mL 0.25 g/mL mixture of Kaolin/normal saline, *n* = 5
3. 2 mL 0.1 g/mL mixture of Kaolin/normal saline, *n* = 5
4. 2 mL 0.005 g/mL mixture of Kaolin/normal saline, *n* = 5.

The operation was limited to <20 min each rat so as to avoid air drying of the organs.

In a second stage, we used a colon abrasion with enterotomy model (*n* = 5) to simulate a colon resection with anastomosis performed at a different site on the caecum to the abrasion. Rats underwent a laparotomy as above followed by a caecum incision to create a full thickness enterotomy over a length of 1 cm away from the abrasion site. The enterotomy defect was then closed with a continuous 4-0 PDS suture (resorbable, monofilament) and the repair leak tested with a simple pressure test. 2 ml 0.005 g/mL Kaolin in saline was instilled over the abrasion (Figure 1D) and sutured site before closure of the abdominal wall. The rats in this group were monitored for 3 weeks as part of the larger experiment protocol.

### Postoperative Monitoring

Post-surgery, the animals were housed individually in separate cages. Animals were monitored postoperatively 8-hourly for the first 48 h to observe their weight, behavior, physical well-being and appearance by using the Clinical Record Sheet, as approved by AEC. Distress scores higher than 6 or weight loss >15% required that animals be humanely killed.

### Outcome Measures

**Figure 2:**
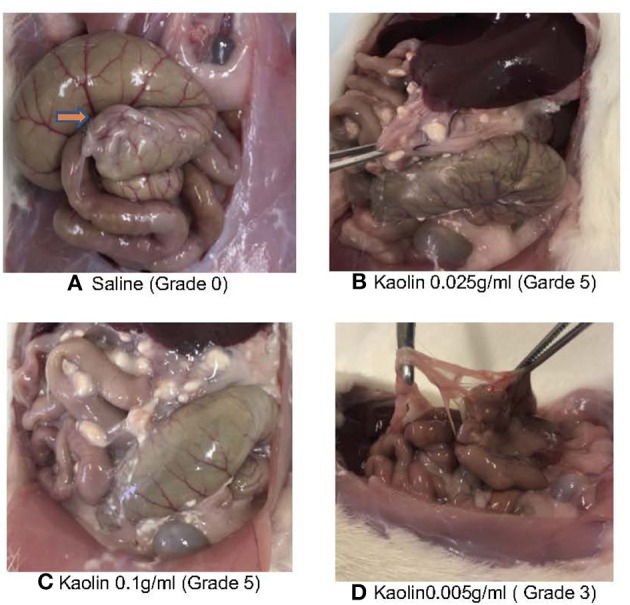
**(A)** Post euthanasia Caecum saline treatment showing minimal or no adhesion (orange arrow). **(B)** Post euthanasia Caecum Kaolin 0.025 g/ml showing Grade 5 adhesion. **(C)** Post euthanasia Caecum Kaolin 0.1 g/ml treatment showing Grade 5 adhesion. **(D)** Post euthanasia Caecum Kaolin 0.005 g/ml treatment showing Garde 3 adhesion.

The animals were humanely killed on postoperative day 14 and scored based on the presence and severity of adhesions using a previously validated adhesion scoring system Table 1 (10). This scoring system takes into account the number, strength and distribution of adhesions formed (Figure 2). Pictures were taken by iPhone 8 12 mp *f* /1.8 aperture camera and also evaluated by a blinded observer.

**Table 1:** Adhesion scoring scheme.

**Adhesion scoring scheme**	
**Adhesion scoring scheme**	**Score description**
0	No adhesions
1	Thin filmy adhesions
2	More than one thin adhesion
3	Thick adhesion with focal point
4	Thick adhesion with planar attachment
5	Very thick vascularized adhesions or more than one planar adhesion

### Histology

**Figure 3:**
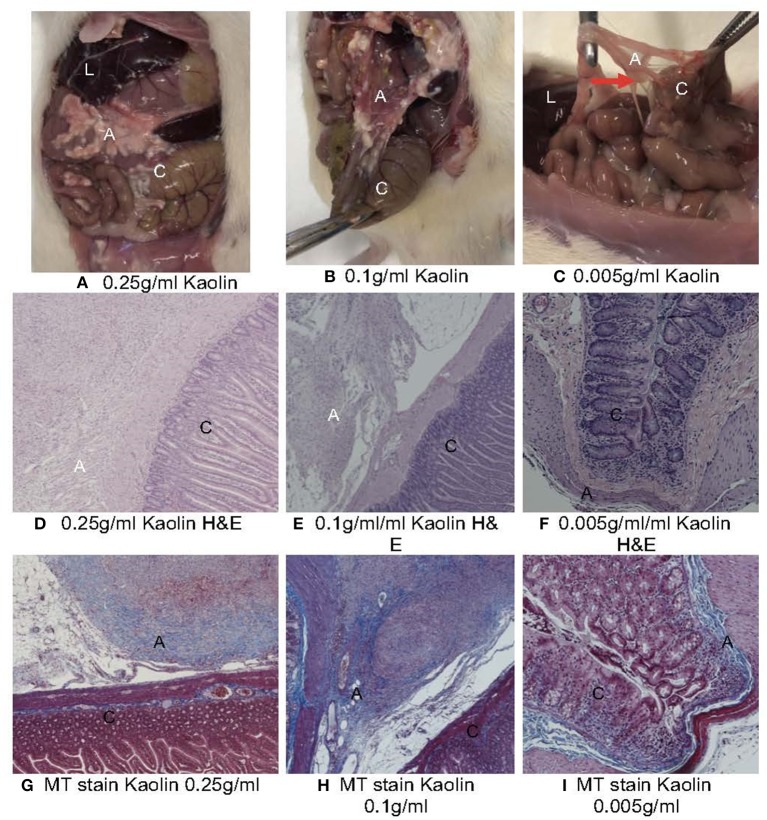
**(A)** Macroscopic **(A–C)** and histopathological **(D–I)** evaluation of abdominal cavity of Rats treated with various concentrations of Kaolin. L, liver; C, caecum; A, adhesion. **(A)** 0.25 g/ml Kaolin causing very thick vascularized adhesions or more than one planar adhesion (Grade 5), **(B)** 0.1 g/ml Kaolin causing very thick adhesions with planar adhesion (Grade 4), **(C)** 0.005 g/ml Kaolin causing thick adhesion with focal point (red arrow) (Grade 3) **(D–I)**. Histopathology of rat caecum, 4X magnification using Haematoxylin & Eosin staining **(D–F)** and Masson's Trichrome staining **(G–I)**. 0.25 g/ml Kaolin treatment showing thick adhesions and polymorphonuclear cell infiltrates **(D)** with disorderly and dense collagen deposition **(G)**. 0.1 g/ml Kaolin with polymorphonuclear cell infiltrates and foreign body reaction **(E)** and disorderly and dense collagen deposition **(H)**. 0.005 g/ml Kaolin with minimal polymorphonuclear cells **(F)** and orderly and light collagen deposition **(I)**.

The caecum, and adhesions between the caecal adventitia and adherent, adjacent intestinal serosal surfaces, and between the adventitial aspect of the caecum and the parietal peritoneum of the abdominal wall, were collected and immersion-fixed in 10% neutral buffered formalin. These tissues were then paraffin-embedded, cut at 6μm, and stained with haematoxylin and eosin (H&E) (Figures 3A–F). Duplicate sections were also stained by the Masson's trichrome technique to demonstrate collagen deposition in fibrous adhesions (Figures 3G–I).

### Statistical Analysis

All statistics were performed using R statistical software (R Foundation for Statistical Computing, Vienna, Austria) through the Jupyter notebook interface. The R package “MASS” (11) was used for ordinal regression. The “polr” function from MASS was used to fit a proportional odds logistic regression model for the ordinal outcome variable (the adhesion score as scored by the primary surgeons), with the treatment as the explanatory variable. A Likelihood ratio test (using the R function “anova”) was used to compare the model with a null ordinal regression model. The means of the ordinal response (interpreted as a numeric value from 1 to the number of classes) were calculated and *post-hoc* pairwise contrasts for each pair of levels of the treatment variable were compared using the “emmeans” package (11), using the Tukey method for *p*-value correction. Statistical significance was taken at the traditional 0.05 level.

## Results

### Adhesion Scores

Control rats receiving colon abrasion and saline and had variable adhesion scores with a mean adhesion score of 1(SD1) and 2/5 having an adhesion score of 0 (no adhesions) (Figure 2A).

Four rats died in the treatment groups with high Kaolin doses, 2 in the group treated with 4 ml 0.25 g/ml and 2 in the group receiving 2 ml 0.1 g/ml Kaolin (Figures 2B,C). Post-mortem evaluation showed severe adhesions with complications of intestinal obstruction, thought to be the likely cause of demise. The remaining rats which lasted the full 14 days showed mean adhesion grades of 4 (SD 0.44) (Figure 3A) and 4.6 (SD 0.6324) (Figure 3B) for 4 ml 0.25 and 2 ml 0.1 g/mL respectively.

Five rats received 2 ml 0.005 g/mL of Kaolin. These rats tolerated the procedure well with no significant morbidity or mortality at the end of 14 days recovery period. The resultant adhesions were mean grade 3.4 SD 0.54 (Figure 2D). The grade of adhesions was significantly greater in the 0.005 g/mL Kaolin treated rats compared to saline treated rats (*p* < 0.0001). Similarly, the abrasion with enterotomy group, treated with Kaolin 0.005 g/ml showed much thicker and vascularized adhesions consistently over the enterotomy site in comparison to the abrasion site. These rats had mean adhesion grade of 4(SD 0.816) and was significantly higher than the Kaolin 0.005 g/ml treated abrasion alone model with adhesion grade 3.4(SD 0.54) (*p* < 0.0001).

### Histopathology

Microscopic analysis of the various grades of adhesions formed in the presence of Kaolin showed classical foreign body (FB) reaction with granular activity at the epicenter of inflammation (Supplementary Figure 1I) which was not seen in the saline treated caecum. The FB reaction was in the form of numerous invading macrophages (Supplementary Figure 1III), which contained phagocytosed kaolin, with active fibrovascular granulation tissue formation with numerous proliferating fibroblasts and supportive micro-vessels (Supplementary Figure 1II). There were mature adhesions with abundant compact collagen and fewer fibroblasts in the 0.1 and 0.25 g/ml kaolin treated rats. The presence of adhesions was predominantly confined to the abrasion site and one rat to the abdominal wall at the suture site (Supplementary Figure 1V). Masson's trichrome stain (MT stain) demonstrated a clear pattern of adhesion formation due to fibroblastic activity at various stages (Supplementary Figures 1VI–IX). The adhesions from the rats that were treated with higher concentrations of Kaolin (0.25 and 0.1 g/ml) showed a very irregular pattern of collagen distribution (Supplementary Figure 1VI) compared to the uniform nature in the lower dosage group of Kaolin 0.005 g/ml (Supplementary Figure 1IX).

### Ordinal Regression Models

**Figure 4:**
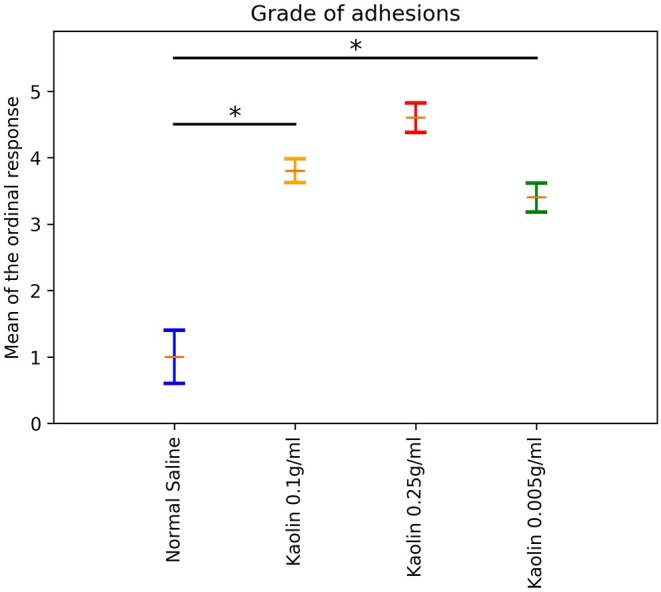
Laparotomy Adhesion score: Bar plot showing different dosages of Kaolin induced adhesion against Ordinal scale in Rats undergone lapratomy and abrasion. The Normal saline treated Rats had minimal, inconsistent adhesion. Rats treated with Kaolin 0.1 g/ml and 0.25 g/ml induced a high grade of adhesion. Rats treated with 0.005 g/ml of Kaolin showed a consistent grade of adhesion between 3 and 4, mean Grade 3.5 (SD 1.475) (*p* < 0.0001). *Statistically significant.

A proportional odds logistic regression model for the ordinal outcome variable was fitted and the means of the ordinal response were calculated as described in the Methods. The means (and standard errors) resulting from fitting the ordinal regression model were plotted in Figure 4. *Post-hoc* pairwise comparisons showed a significantly higher mean adhesion grades for Kaolin doses 0.1 g/ml and 0.005 g/ml compared to normal saline (*p* < 0.0001; Figure 4).

Another ordinal model was fitted to compare the adhesions caused by Kaolin 0.005 g/ml in the Laparotomy model with abrasion vs. the same dose in the abrasion with enterotomy model. The Enterotomy model showed a higher mean ordinal response (difference in mean ordinal response = 1.3; *p* < 0.0001).

## Discussion

We present for the first time, a small animal model capable of consistent non-lethal adhesion formation within the abdominal cavity of rats. Kaolin administered at a low concentration of 0.005 g/ml following colon abrasion and enterotomy, produced consistent moderate to severe grade adhesions with a uniform distribution of collagen fibers on microscopic examination.

Creating an animal model for abdominal adhesions with consistent, reliable and reproducible findings for the positive control is a challenge. Unlike humans, where adhesions are almost inevitable after abdominal surgery, in the rat model, no or only low-grade adhesions are commonly found after laparotomy alone. Also, in this study, no or limited adhesions were found in the saline control animals. Several types of animal models have been used, small (mice, rat and rabbit) and large (sheep, pig, monkey and horse) (12). Models do stimulate adhesion-formation in different ways, including colon and side wall abrasion, crushing, desiccation, incision, excision, electrocautery, laser injury, thermal injury, chemical injury, radiation injury, and foreign body-tissue irritation (12, 13). However, the usefulness of those models is hampered by the variability of adhesion formation in the positive control animals. This reduces the power of those studies increasing the number of animals that is required to test the anti-adhesive properties of test compounds and takes a longer period to replicate. Indeed, the strength of a model lies in the ability to replicate a similar injury process as in human conditions producing similar uniform non-lethal forms of adhesions in positive control animals. Kraemer et al. (14) compared 5 different types of injury models and demonstrated good adhesion formation but they were performed on the parietal wall of the abdomen which does not mimic the laparotomy model and does not cause the serosal or mesothelial injury. diZeerga et al. describes that clean-cut incisional wounds are not enough to stimulate fibrin deposition and in contrast, cautery and thermal injury causes excessive tissue necrosis with formation of mature fibrotic bands after more than 21 days (12, 13). Ozel et al. (14) discusses the chemical injury model using alcohol and iodine which are inherently disinfectants and are not suitable for an infective (enterotomy) model. Hence a chemical which is potent enough to create a foreign body reaction at the site of mechanical injury caused by abrasion and limited in its role as a general irritant is ideal. Kaolin or commonly called “chalk,” is a mixture of different minerals and is a naturally occurring aluminum silicate mineral derived from clay. It contains quartz, mica, feldspar, iolite and montmorillonite. Kaolin is used in paper production, in paints, rubber, plastic, ceramic, chemical, pharmaceutical and cosmetic industries^1^. Pairon et al. (15) in 1990 studied the interaction of Kaolin with cell lines and found a variety of membrane interactions and metabolic impairments. Kaolin in the recent past has been of interest in clinical studies due to its role in achieving haemostasis in Oculoplastic Surgery as a local application (16) or intra-abdominal surgery with Kaolin impregnated gauze as a leave-in substance for rapid hemorrhage control in critically injured patients in combat (17).

Kaolin has the universal property of causing a foreign body reaction and inducing an inflammatory response that induces adhesion formation (18, 19). The injury is similar to the mesothelial injury in abdominal adhesion by foreign body reaction and setting up a wound healing process resulting in fibrosis/adhesion as seen in pulmonary fibrosis (20).

The pathology thus generated could replicate the human condition of tissue handling, glove powder, mechanical injury due to clamps and electrocautery. A rat model is relatively easy to use and replicate in terms of the experiment and also the ratio of the peritoneal surface area relative to the body weight and height is comparable to human (12). The volume of adhesion inducing agent also matters when we test an anti-adhesive substance, hence refinement of 4 mL to 2 mL is significant in terms of animal discomfort post-surgery. The surface area in the rat abdomen is high but the volume of chemical used to induce injury has to be titrated sufficient enough to cause injury and provide space for the anti-adhesive agent. The dosage of Kaolin that's ideal in both the laparotomy with abrasion alone and abrasion with enterotomy model was 0.005 g/ml and this produces consistent adhesion without being harmful to the rat. Interestingly as expected there was higher grade of adhesion seen with the enterotomy model in compared to the laparotomy with abrasion alone group, but the rats were able to tolerate the insult and recovered without any morbidity or weight loss.

One of the limitations in this model is the procoagulant property of Kaolin which may inhibit adhesion formation (17, 21). In spite of which, the overall ability of its property to induce chemical injury has resulted in a controlled amount of adhesion formation using a low dosage.

In conclusion, the purpose of this study was to make a reliable rat model of adhesion formation. This has been achieved by application of a low dosage of Kaolin silicate (0.005 g/mL) to the site of peritoneal injury, causing a consistent non-lethal adhesion in positive control animals. The model is safe and efficacious and can now be used in future studies to assess anti-adhesive properties of test compounds for abdominal adhesion prevention experiments.

## Data Availability Statement

The datasets generated for this study are available on request to the corresponding author.

## Ethics Statement

The animal study was reviewed and approved by The University of Adelaide and Central Adelaide Local Health Network Animal Ethics Committee SA Pathology Animal Ethics Committees (AEC).

## Author Contributions

RV, CB, AB, MS, and JF were involved in the designing and conduct of the study, collection of data, analysis, interpretation of results, drafting, and correction of the manuscript. MT, AP, SV, and PW were involved in designing the experiments, supervision, interpretation of results, correction, and final approval of the manuscript.

### Conflict of Interest

PW is on the patent for Chitogel and combination therapy. SV is on the patent for combination therapy. The remaining authors declare that the research was conducted in the absence of any commercial or financial relationships that could be construed as a potential conflict of interest.

## Footnotes

^1^https://toxnet.nlm.nih.gov/cgibin/sis/search2/r?dbs+hsdb:@term+@DOCNO+630

## Supplementary Material

Supplementary Figure 1 Illustrates the temporal development of adhesion. **(I)** Formation of an active adhesion projecting from the caecal adventitial surface and comprised of fibrovascular granulation tissue and collagen deposition (arrow) H&E. **(II)** Higher power view of a similar adhesion to that shown in this figure. There is robust fibrovascular proliferation and invading macrophages containing phagocytosed administered exogenous material (arrow) H&E. **(III)** An early adhesion showing active fibroblastic proliferation, with loosely arranged collagen fibrils evident. Numerous micro-vessels are present (arrow) H&E. **(IV)** More mature adhesions showing abundant collagen deposition. H&E. **(V)** Adhesion projecting from the abdominal wall (a-abdominal wall) H&E. **(VI,VII)** Adhesion composed of numerous bundles of collagenous connective tissue, admixed with invading macrophages (arrows). Masson's trichrome. **(VIII)** Well-developed fibrous adhesion between two loops of bowel. Masson's trichrome (A in box Adhesion, C- caecal serosal wall). **(IX)** Diffuse adhesion projecting from the caecal serosal surface into the mesentery (A in box Adhesion, C- caecal serosal wall).
