# Supplementary figures and images for: A Novel Rat Model to Test Intra-Abdominal Anti-adhesive Therapy
Source: Front Surg. 2020 Apr 8;7:12. doi: 10.3389/fsurg.2020.00012 (PMC7158702; doi:10.3389/fsurg.2020.00012)

Supplementary Figure 1:

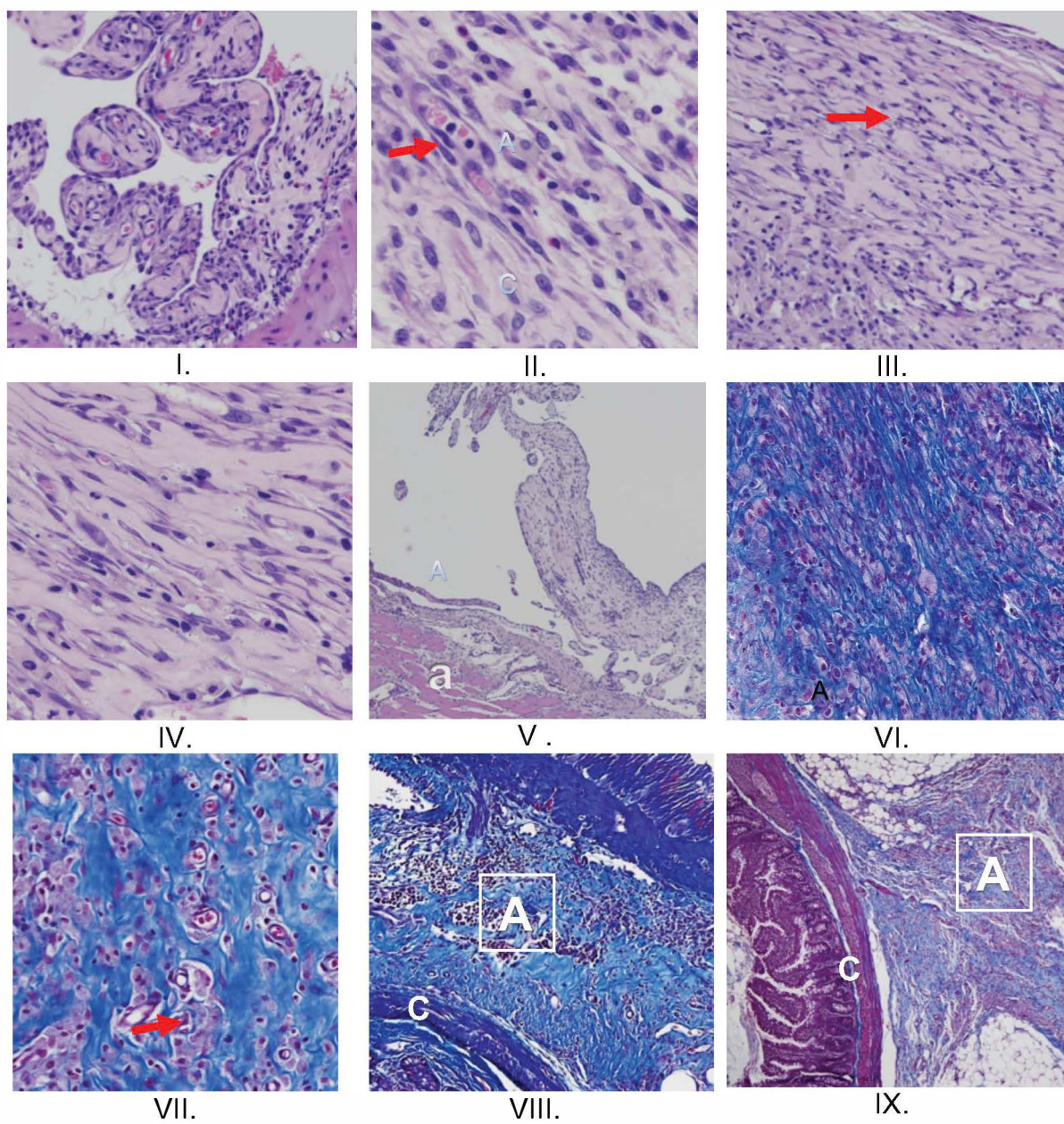

Supplement: Supplementary Figure 1 — Illustrates the temporal development of adhesion. (I) Formation of an active adhesion projecting from the caecal adventitial surface and comprised of fibrovascular granulation tissue and collagen deposition (arrow) H&E. (II) Higher power view of a similar adhesion to that shown in this figure. There is robust fibrovascular proliferation and invading macrophages containing phagocytosed administered exogenous material (arrow) H&E. (III) An early adhesion showing active fibroblastic proliferation, with loosely arranged collagen fibrils evident. Numerous micro-vessels are present (arrow) H&E. (IV) More mature adhesions showing abundant collagen deposition. H&E. (V) Adhesion projecting from the abdominal wall (a-abdominal wall) H&E. (VI,VII) Adhesion composed of numerous bundles of collagenous connective tissue, admixed with invading macrophages (arrows). Masson's trichrome. (VIII) Well-developed fibrous adhesion between two loops of bowel. Masson's trichrome (A in box Adhesion, C- caecal serosal wall). (IX) Diffuse adhesion projecting from the caecal serosal surface into the mesentery (A in box Adhesion, C- caecal serosal wall). [file Image_1.pdf]
